# Cellular Aging Characteristics and Their Association with Age-Related Disorders

**DOI:** 10.3390/antiox9020094

**Published:** 2020-01-22

**Authors:** Magdalena Rudzińska, Alessandro Parodi, Anastasia V. Balakireva, Olga E. Chepikova, Franco M. Venanzi, Andrey A. Zamyatnin

**Affiliations:** 1 Institute of Molecular Medicine, Sechenov First Moscow State Medical University, 119991 Moscow, Russia; magdda.rudzinska@gmail.com (M.R.); aparodi.sechenovuniversity@gmail.com (A.P.); balakireva.anastacia@gmail.com (A.V.B.); chepikovaolga@gmail.com (O.E.C.); francovenanzi51@gmail.com (F.M.V.); 2 Belozersky Institute of Physico-Chemical Biology, Lomonosov Moscow State University, 119991 Moscow, Russia

**Keywords:** aging hallmarks, oxidation, protein carbonylation, age-related diseases

## Abstract

Different molecular signaling pathways, biological processes, and intercellular communication mechanisms control longevity and are affected during cellular senescence. Recent data have suggested that organelle communication, as well as genomic and metabolic dysfunctions, contribute to this phenomenon. Oxidative stress plays a critical role by inducing structural modifications to biological molecules while affecting their function and catabolism and eventually contributing to the onset of age-related dysfunctions. In this scenario, proteins are not adequately degraded and accumulate in the cell cytoplasm as toxic aggregates, increasing cell senescence progression. In particular, carbonylation, defined as a chemical reaction that covalently and irreversibly modifies proteins with carbonyl groups, is considered to be a significant indicator of protein oxidative stress and aging. Here, we emphasize the role and dysregulation of the molecular pathways controlling cell metabolism and proteostasis, the complexity of the mechanisms that occur during aging, and their association with various age-related disorders. The last segment of the review details current knowledge on protein carbonylation as a biomarker of cellular senescence in the development of diagnostics and therapeutics for age-related dysfunctions.

## 1. Introduction

The regulation of signaling pathways, protein homeostasis (proteostasis), organelle-organelle communication, and organelle-cytosol communication is essential for proper cellular functioning [1]. The molecular signals activated by secreted metabolites, lipids, proteins, and nucleic acids initiate transduction cascades in different cellular compartments, regulating several fundamental processes, such as cell proliferation, apoptosis, proteolysis, and autophagy [2,3]. Aging can affect these phenomena at different levels, impacting cell fitness, size, and molecular mechanisms while decreasing division capability [4,5]. The incidence of age-related disorders, such as atherosclerosis, hypertension, type 2 diabetes, osteoporosis, cataracts, Alzheimer’s, and cardiovascular disease, increases exponentially with age progression through mechanisms that are still not fully understood [6].

Few discrete hallmarks can be directly connected to the aging process, including chromosome structure, epigenetic regulation, telomere attrition, the loss of proteostasis, energy metabolism, mitochondrial dysfunction, and altered intercellular communication [4,7]. The detection of quantitative age-associated biomarkers can allow for measuring aging degeneration and predicting the lifespan of individual organisms [8,9]. The main aim of aging research is to dissect the connections between aging hallmarks and their relative contribution to pathological conditions [7].

Herein, we describe several crucial processes responsible for the regulation of cellular aging, including the essence of carbonylation, its role in perturbing proper protein folding, and its potential value as an aging biomarker.

## 2. Characteristics of the Senescent Phenotype

Due to the accumulation of DNA damage and telomere erosion, cells irreversibly arrest in the G1 phase of the cell cycle, and this phenomenon is considered a symptom of replicative senescence [7,10]. In yeast such as *Saccharomyces cerevisiae*, reproduction occurs through the budding of the daughter cell from the mother cell. In this phenomenon, the aged and damaged cellular material accumulates in the mother cell, and after more than 26 division cycles, expresses apoptotic and degeneration signs, including high levels of reactive oxygen species (ROS), DNA fragmentation, and protein carbonylation [11]. The target of rapamycin (TOR) signaling, dietary restriction, and pH regulate this asymmetric division of the cellular material in yeast [11,12,13]. 

Similarly, it has been shown that mammalian stem cells segregate new mitochondria to daughter cells, affecting their stemness potential [14]. DNA damage, proteomic deregulation, and senescence-associated secretory phenotypes are typical characteristics distinguishing between old and nonsenescent cells [15]. The most common aging biomarkers are considered to be β-galactosidase labelling at pH 6.0 and the decreased expression of tumor-suppressor proteins (such as cyclin-dependent kinase inhibitor 2A and p53 [16].

Furthermore, aging is associated with cytoskeletal disorganization and changes in nuclear integrity, leading to severe impairments of the cellular structure in different organisms such as worms, yeast, *Drosophila*, and humans [17,18,19,20]. In yeast, ROS accumulation and Ras/cAMP (proto-oncogene, GTPase/cAMP-dependent protein kinase) signaling pathway activation are at the root of actin turnover and cytoskeleton destabilization [21]. In humans, the disruption of the cytoskeleton structure can be a result of Tau microtubule-associated protein hyperphosphorylation, which is also associated with the generation of neurofibrillary tangles and proteasome inhibition in Alzheimer’s patients [22]. 

In addition, the oxidative degradation of nucleoporins (the proteins of the nuclear envelope that regulate the exchange of molecules between the nucleus and the cytosol) contributes to the deregulation of proper cellular functioning. Differences in nuclear envelope protein organization (e.g., nuclear lamins) and nuclear architecture have been observed in Hutchinson-Gilford progeria patients, cardiomyopathies cases, and damaged stem cells [23]. 

### 2.1. Genome Maintenance and Epigenetic Mechanisms

Genome instability refers to a variety of DNA alterations induced by intra- and extracellular factors, such as DNA replication errors, ROS, and exogenous insults (e.g., ultraviolet irradiation) [24,25]. Somatic mutations accumulate within the cells in all organisms, inducing the activation of age-related signaling pathways and consequent cell disorders, senescence, and death. DNA double-strand break (DSB) is one of the most severe kinds of damage, and it is repaired by two mechanisms: homologous recombination (HR) and nonhomologous end-joining (NHEJ). 

While HR requires the presence of a homologous sequence to repair the DNA, the NHEJ mechanism allows for ligating the broken ends without the presence of a homologous template. Both of these processes are crucial for genome stability and cooperate with non-DSB repair mechanisms [26] such as base excision repair [27], nucleotide excision repair [28], and mismatch repair [29].

Telomere length is inversely correlated with age, and the ends of the chromosomes are protected from degradation and incorrect recombination by telomere caps (nucleoprotein complexes). Telomerase enzymes prevent their shortening [30], which is at the root of different pathological conditions such as telomere-mediated syndromes (e.g., dyskeratosis congenital and idiopathic pulmonary fibrosis) and Alzheimer’s disease [31,32,33]. Chromatin structure alteration occurring during aging can lead to changes in the gene expression pattern and can affect an organism’s lifespan. Aging is also related to epigenetic mechanisms such as DNA chemical modifications (e.g., methylation), histone modifications (such as acetylation/deacetylation), and post-transcriptional gene regulation induced by noncoding RNA [34,35]. 

The process of gene methylation is significantly affected during aging and occurs via a transfer of a methyl group from *S*-adenosyl-*L*-methionine to the carbon in position 5 of the cytosine (C-5; CpG sites) by the enzyme DNA methyltransferase [36]. In particular, the altered methylation of three genes—*EDARADD* (Edar-associated death domain), *TOM1L1* (Target of Myb1-like 1 membrane-trafficking protein), and *NPTX2* (Neuronal pentraxin II)—have been frequently registered in the elderly [37,38]. The proteins encoded by these transcripts play different functions. *EDARADD* is required for the development of hair, teeth, and other ectodermal structures [39]; *TOM1L1* acts as an adapter protein involved in several signaling pathways [40]; and *NPTX1* can be involved in synaptic scaling [41].

Histones are reversibly acetylated and deacetylated by the action of histone/lysine acetyltransferase (HAT/KAT) and histone deacetylase (HDAC) enzymes, respectively [42]. Gene transcription is associated with increased histone acetylation, which induces a more relaxed chromatin structure, whereas histone deacetylation is related to more condensed DNA and reduced transcription [34]. It has been shown that the downregulation of HDACs (such as Sirtuin2, SIR2, and HDAC1) is involved in the extension of the lifespan of yeast (*Caenorhabditis elegans* [43,44] and *Drosophila* [45]). In human cells, histone acetylation decreases during aging, and this phenomenon is directly related to a reduced cell metabolic rate and proliferation [46].

### 2.2. RNA Maintenance and Protein Synthesis

Recent data from a big RNA meta-analysis performed on young and old murine, rat, and human specimens allowed for characterizing the age-related patterns of gene expression, defining the role of different genes involved in inflammation, the immune response, and lysosomal degradation [47]. However, the analysis demonstrated that aging occurs through several pathways in various tissues and species and that it does not depend on a universal molecular program [48].

RNA maintenance (i.e., ribostasis) is a process that is not yet universally accepted as a hallmark of aging, but growing evidence has suggested its involvement in this phenomenon. In prokaryotes (e.g., parasites), self-splicing mobile introns might play a regulatory role in gene expression and have evolved to respond to environmental conditions, such as ROS, temperature, and starvation [49]. Their deletion in the mitochondrial genome of *Saccharomyces cerevisiae* results in harmful consequences for cells [50]. In eukaryotes, pre-mRNA (including exons separated by introns) splicing is a fundamental link between gene expression and the proteome. Alternative splicing defects can arise when the levels or functions of generic spliceosome components are altered [51]. Splicing alterations can occur to genes belonging to pathways related to aging (e.g., DNA repair genes), ultimately accelerating this process [52]. Mechanistically, aberrant splicing leads to aging-related phenotypes through decreased or increased isoform function and an imbalanced isoform ratio [51]. As an example, splicing defects occurring in tumor protein p53, insulin-like growth factor IGF-1, and Sirtuin 1 (*SIRT1*) genes are associated with progeria, vascular aging, and Alzheimer’s disease [53]. 

During aging, protein translation globally decreases [54], affecting the expression of the selective proteins required for cellular maintenance [55], while cysteine misincorporation increases [56]. Additionally, proteome studies have revealed differences in protein composition and the upregulation of proteins involved in energy metabolism, proteostasis, the cell cycle, the response to stress-signal transduction, and apoptosis [57,58,59], which are mainly regulated by post-transcriptional mechanisms [59].

The translation process is also regulated by non-protein-coding RNAs (ncRNAs), which include miRNA (approximate length of 21–23 nucleotides) and lncRNA (approximate length ≥ 200 nucleotides): ncRNAs regulate a wide range of biological processes, including metabolism and aging [60,61], affecting chromosome structure, transcription, splicing, mRNA stability and availability, and post-translational modifications [62]. 

When miRNAs base-pair with their target mRNAs at 3′UTR, this leads to mRNA degradation and/or translational repression [63]. Many targets of miRNAs are implicated in aging and modulate longevity [64], and for this reason, circulating miRNAs could provide a reliable and easy way to measure aging progression [65]. In particular, Hooten et al. have demonstrated that the three miRNAs 151a-5p, miR-181a-5p, and miR-1248 significantly decrease during human aging [66]. 

Additionally, lncRNAs have been shown to contribute to neuronal pathogenesis [67] through the modulation of gene expression in the central nervous system [68].

## 3. Mitochondria

Mitochondria and metabolic activity, including nutrient-sensing mechanisms, are connected with lifespan and aging [69]. It has been shown that mtDNA mutations increase with age, and the number and type of mutations are tissue- and cell phenotype-specific [70]. A decrease in the activity of mitochondrial enzymes (e.g., citrate synthase) and the accumulation of mtDNA mutations are associated with a decline in mitochondrial respiratory efficiency [71]. Mitochondria are one of the most important sources of ROS, and mitochondrial dysfunctions are considered to be a primary cause of mitochondrial oxidative-dependent damage [72]. However, many studies have indicated that mtDNA alteration could be a result of ROS-independent phenomena such as replication errors and failure of the repair mechanisms [73,74,75]. In this context, mice with mtDNA polymerase gamma knock-down (PolgD257A/D275A) have shown somatic mtDNA mutation accumulation and shorter longevity without increasing ROS production [76]. 

Mitochondrial dysfunctions are associated with neuronal degeneration in age-related diseases, such as Alzheimer’s or Type 2 diabetes [77]. In an Alzheimer’s murine model and in the temporal neocortex of patients with Alzheimer’s disease, a higher expression of mitochondrial deacetylase SIRT3 was observed [78]. In this context, SIRT3 acted as a neuroprotective molecule and mitigated stress condition effects. Moreover, the investigation showed that the downregulation of SIRT3 decreased the activity of metabolic enzyme complex I and ATP levels [67].

## 4. Metabolic Activity

Dietary restrictions and hormone levels can strongly affect longevity in all organisms [79]. Metabolism in mammalians is regulated at multiple levels by hormone actions, and intricate feedback [80]. The somatotropic axis of insulin-like growth factor 1 (IGF-1)/growth hormone (GH); the Notch, transforming growth factor β (TGF-β), and WNT pathways; and their interplay control the regulation of many processes, including the development of a senescent phenotype (Table 1) [81]. 

IGF-1/insulin signaling (IIS) is considered to be an aging-controlling pathway, and among its targets are the Forkhead box (FOX) family and the mammalian target of rapamycin kinase (mTOR) complexes [82].

Stimulation of the insulin receptor extends the life of worms and mammals via the activation of the phosphoinositide 3-kinase (PI3K)/dependent-protein kinase B (AKT)/mTORC2 (PI3K/AKT/mTORC2) signaling pathway and the inhibition of the phosphorylation of forkhead box protein O (FOXO) transcription factor [83]. Whereas mTORC2 is involved in IGF–PI3K signaling, which promotes cell proliferation and regulates oxidative stress, mTORC1 promotes protein translation, ribosome biogenesis, and autophagy [84]. Both TORC1 and TORC2 are effectors in pathways connected by caloric restriction (the restriction of food intake without malnutrition), which prolongs the lifespan of various species [85]. The critical intracellular signals for TORC1 activation, which can delay aging, are dietary regimens based on a reduced intake of amino acids [86]. The activation of TORC1 occurs on the lysosome surface in mammalian cells and in vacuoles in yeast cells, and it leads to the phosphorylation of target proteins activating or inhibiting pro- or antiaging processes, respectively [84,87].

While IIS is involved in the activation of pathways related to nutrient abundance/anabolism, the nutrient sensors AMPK and Sirtuins are induced by nutrient deficiency/catabolism. AMP-activated protein kinase (AMPK) is an energy-sensing enzyme. In mammalian cells, AMPK phosphorylates FOXO and inhibits mTOR [88], thus controlling energy metabolism, autophagy, stress resistance, and ultimately the aging process [89]. The Sirtuins family of nicotinamide-adenine dinucleotide (NAD)-dependent protein deacetylases has been identified as a regulator of the replicative lifespan in yeast, as described above [90,91]. Here, SIR2 proteins can also mediate the beneficial effects of dietary restriction on longevity in yeast, worms, flies, and mice [92].

## 5. Interorganellar Communication

Dynamic changes in organelle communication are essential for biological systems and are involved in cellular aging [1]. Organelles perform specific functions in a single cell, but globally they play in concert. In intracellular communication, the physical interaction between organelles is not required, and information exchange occurs through soluble molecules. In this scenario, organelle-organelle and organelle-cytosol communications are based on metabolites such as lipids, proteins, peptides, or nucleic acids, which can function as pro- and antiaging signals [111].

Cellular aging is a process integrated with metabolism and proteostasis and their mutual interactions. Thereby, all metabolic disorders that affect proteostasis can enhance aging via feed-forward connectivity [112]. In stress conditions, the misfolded/damaged proteins accumulate in aggregates, which are dispersed in the cytosol or are associated with the endoplasmic reticulum (ER), mitochondria, and/or vacuoles affecting their functioning [113].

Next, protein homeostasis in mitochondria is sustained by mitochondria-to-nucleus communication, inducing antiaging unfolded protein response pathways. The unfolded and/or misfolded proteins in the intermembrane space of mitochondria mediate AMPK kinase (SNF1 in yeast) cascade activation, which initiates a quality control via proteases and chaperone activity [113,114,115].

Direct physical interaction between different organelles occurs at membrane contact sites, including vacuole-mitochondria patches, mitochondria-associated ER membranes, and the perinuclear ER-vacuolar junctions. Membrane contact sites are essential for ion (e.g., Ca^2+^) and lipid homeostasis, supporting many functions such as metabolism, apoptosis, and organelle dynamics [116,117].

The ER represents central intracellular Ca^2+^ storage and is crucial for Ca^2+^ homeostasis [117,118]. Multiple sites near the plasma membrane (PM) and ER-PM contact sites are critical for Ca^2+^ ER refilling and maintenance [118]. The nuclear envelope, which is in continuity with the ER, can communicate with the PM, carrying signals from the extracellular space into the nucleus [118]. In addition, peroxisomes and the Golgi apparatus are known to uptake or release Ca^2+^ in response to agonists [119,120]. 

Alterations in ER loading and the release of Ca^2+^ are associated with aging. For example, in aged mice, stimulation with a neurotransmitter (acetylcholine) significantly increased the intracellular release of Ca^2+^ from the ER [121]. Furthermore, aged cells with an interrupted Ca^2+^ balance demonstrated an impaired ER response to stress conditions [122]. 

The ER-mitochondria interface regulates the flux of metabolites between these organelles and is fundamental for many cellular processes, including cell proliferation, death, autophagy, calcium signaling, unfolded protein response, and inflammation [123]. A decrease in mitochondrial Ca^2+^ uptake and a lower number of mitochondria-ER connections were associated with cellular senescence in vitro (cell passage-dependent). Furthermore, the structure of mitochondria-ER patches resulted in enriched proteins associated with age-related neurological and metabolic disorders [124].

Next, it has been shown that perturbations in these pathways through the silencing of the mitochondrial fusion proteins Mitofusin-1 (Mfn1) and -2 caused alterations in Ca^2+^ signaling as well as in the ER and in mitochondrial dysfunctions [125,126]. The downregulation of Mfn1 and Mfn2 was linked with cardiac and skeletal muscle hypertrophy in rats and patients with type 2 diabetes or obesity, respectively [127,128]. On the other hand, Alzheimer’s disease has been recently related to an increase in ER-mitochondria contact. Such increments can explain many of the biochemical and morphological changes that affect dopaminergic neurons in this disease [129]. 

## 6. Proteostasis

**Figure 1:**
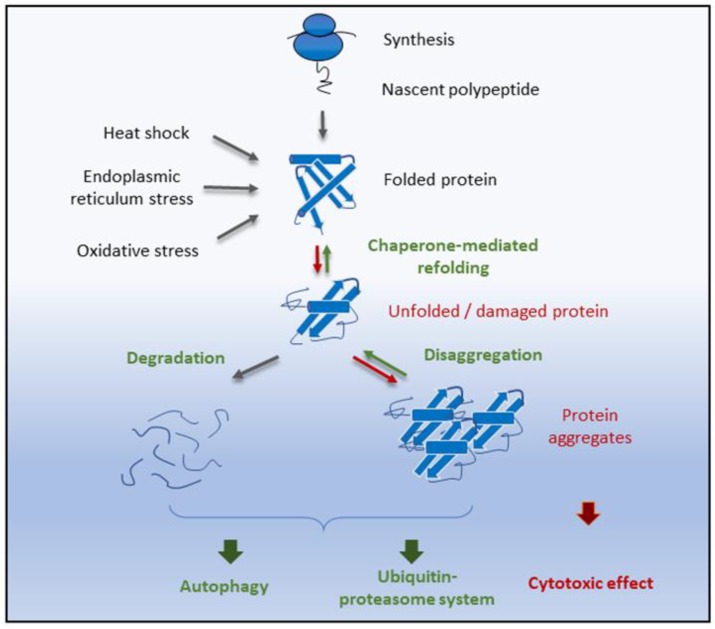
Pathways of proteostasis under stress conditions. Under normal conditions, misfolded proteins are degraded and/or cleared extracellularly, undergo autophagy, or are degraded with the aid of the cellular proteasome. Endogenous and exogenous stress conditions contribute to cellular dysfunction, protein misfolding, and protein aggregations that lead to the loss of homeostasis. Unfolded and damaged proteins are refolded by heat shock proteins through the chaperone-mediated folding pathway, or they are destructed by ubiquitin–proteasome or autophagic pathways (micro- and macroautophagy). Deficiencies in refolding or degrading unfolded proteins can lead to the formation of toxic aggregation.

As was underlined above, protein homeostasis, or proteostasis, refers to the regulation of protein synthesis, folding, and unfolding and turnover of the proteins. Proteostasis is critical for the longevity of cells and the functioning of organisms [130,131]. It is regulated by quality control systems integrating chaperon and protein degradation pathways, having a significant impact on aging degeneration [132,133]. In healthy cells, tissues, or organisms, a balanced proteome is maintained by a proteostasis network that regulates three steps of protein life: protein synthesis and folding, conformational maintenance, and degradation (Figure 1). The critical regulators of those steps are the chaperones that ensure proper de novo folding of a newly synthesized protein or the recognition of misfolded proteins [134]. Degradation pathways include the ubiquitin–proteasome system (UPS) and the autophagic lysosomal-endosomal pathway [135]. The proteostasis machinery is affected by stress conditions, such as elevated temperature or oxidative stress (Figure 1). Under stress conditions, the unassembled proteins can aggregate, becoming terminally misfolded or nonfunctional. In these conditions, they are toxic, and they need to be degraded to avoid damage [136]. Age-related diseases, such as Parkinson, Alzheimer’s, and amyotrophic lateral sclerosis, are connected with errors in the assembly of proteins, reduced chaperoning, proteasomal and autophagic activities, and the accumulation of toxic aggregates in the cells [137,138]. 

The proteasome is a multi-subunit complex that operates mainly in the cytosol and nucleus [139] and is responsible for the rapid degradation of a single protein tagged by ubiquitins. UPS is activated in response to endoplasmic reticulum stress, where environmental and endogenous factors lead to the accumulation of misfolded and unfolded proteins in the ER lumen and eventually to reticulum stress and ER structural damage [140]. UPS mitigates ER stress, restores homeostasis, and promotes cell adaptation to stress conditions. However, under unresolvable stress, UPS promotes apoptosis and death of the cell [141]. Deregulation of the UPS mechanism is connected with diabetes and neurodegeneration [142,143].

Whereas UPS contributes to proteostasis via the proteasome by degrading single proteins, autophagy degrades protein aggregates (microautophagy [144]), membrane-associated proteins (macroautophagy [145]), and specific proteins (chaperone-mediated autophagy (CMA)) [146]. Microautophagy is a direct degradation of intracellular proteins and organelles by lysosome protrusions or by the vacuoles in yeasts [147,148]. In contrast, macroautophagy is an in-bulk degradative pathway that removes superfluous and damaged organelles, such as mitochondria (mitophagy) and ER vesicles (ER-phagy), cytosolic proteins, and invasive microbes, to adapt to stress conditions and to maintain cellular homeostasis. Macroautophagy requires the formation of double or multiple membrane vesicles (autophagosomes), which then fuse with lysosomes or late endosomes for bulk degradation [145,147]. Finally, CMA refers to a particular form of autophagy during which soluble cytosolic proteins are bound to HSC70 chaperone and internalized into lysosome lumens for degradation [149].

If the proteostasis network fails, the formation of pathological protein aggregates occurs. Those aggregates can be divided mainly into two groups: amyloid fibrils and amorphous aggregates [150]. Amyloid-like fibrillar aggregates are recognized as hallmarks of neurogenerative diseases (e.g., Alzheimer’s disease), while amorphous nonfibrillar aggregates of α-crystallin are associated with cataracts [151].

Protein aggregates possess cytotoxic properties: fibrillar aggregates are linear self-assemblies that can interact with the membranes affecting their ultrastructure, while amorphous aggregates are structures without ordered intermolecular interactions and may form pores in membranes [152,153]. Aging and external stress cause the accumulation of these aggregates, and a reduction in the degradation pathway capacity of the proteostasis network can eventually induce an overall failure of the proteostasis process.

## 7. Protein Carbonylation as an Aging Biomarker 

Protein carbonylation is an irreversible and unrepairable oxidative post-translational modification that yields a reactive carbonyl moiety, such as an aldehyde, ketone, or lactam in a protein. These protein modifications accumulate during the life of all organisms [154,155]. Reactive carbonyls can generate from endogenous (e.g., mitochondria, phagocytic) or exogenous (e.g., cigarette smoke, food additives) sources [156], and oxidation may induce both structural and functional alterations of proteins. Collectively, ROS increase the formation of different free radicals and nonradical oxygen derivatives (ONOOH) [157]. The primary protein carbonylation mechanism involves the metal-catalyzed oxidation of amino acid side chains, especially proline, arginine, lysine, and threonine. However, carbonyl derivatives can also be generated through the α-amidation pathway or through the oxidation of glutamyl side chains, where the peptide is blocked in N-terminal amino acids by an α-ketoacyl derivative [158,159]. The secondary mechanism involves the carbonylation of lysine, cysteine, and histidine, which may be caused by their reaction with reactive carbonyl groups produced during the oxidation of carbohydrates (e.g., glyoxal (GO), methylglyoxal (MGO)) and lipids (e.g., 4-hydroxynonenal (4-HNE), malondialdehyde (MDA), acrolein (ACR)). These processes of carbonyl generation are termed glycoxidation (the formation of advanced glycation end products (AGEs)) and lipoxidation (the formation of advanced lipid peroxidation end products (ALEs)) [158,159]. 

Experimental data from plasma, sera, and tissues have suggested a positive correlation between protein carbonyl levels and age [160,161]. Furthermore, carbonylated protein contents dramatically increased in the late phase of life [162], and protein carbonylation was detected especially in the heart, muscle, brain, and plasma of the elderly [163,164,165,166]. 

Carbonylated proteins are degraded through the proteasomal system, but they can avoid degradation and create cytotoxic aggregates [167]. In this context, inducing carbonyl stress in young mice increases protein aggregation similarly to what occurs in physiological conditions in old mice. Furthermore, in one study, over 90% of a carbonylated proteome was present in aggregates collected from the spleen protein lysates of aged mice [168]. These remarks have confirmed that post-translational oxidative alterations (including carbonylation) are responsible for increased protein aggregation.

Many studies have highlighted the relationship between an increase in protein carbonylation and several age-related disorders. Thus, elevated levels of protein carbonyls have been observed in diabetes [169], Parkinson’s disease, Alzheimer’s disease [170], Huntington’s disease [171], amyotrophic lateral sclerosis [172], cancers [173], cataractogenesis [174], Werner syndrome [175], cystic fibrosis [176], and essential arterial hypertension [177]. However, carbonylated proteins have been observed in both the healthy and diseased parts of the brain in patients with Parkinson’s. Additionally, brain regions from individuals with incidental Lewy body disease (putative presymptomatic Parkinson’s disease) showed no increase in carbonyls in any brain areas. It can be speculated that oxidative protein damage can occur widely in the brain, but this phenomenon is evident only in old patients [178]. 

High carbonyl levels have been detected in β-actin, creatine kinase BB, glutamine synthase, and ubiquitin carboxy-terminal hydrolase L-1 in Alzheimer’s patients compared to a control group [179]. In cases of Huntington’s disease, Túnez I. et al. showed a close correlation between global oxidative stress, protein carbonylation, and disease severity. This correlation may indicate that oxidative stress accompanied by protein carbonylation is associated with Huntington’s disease evolution [171]. 

Next, high protein carbonyl levels have been found in both the spinal cord and motor cortex of patients with sporadic amyotrophic lateral sclerosis [172,180]. Although the exact mechanism by which ROS induce cell death in amyotrophic lateral sclerosis is not known yet, the data indicated that carbonylation affected spinal cord cellular proteins, including neurofilaments [135]. 

In the context of cancer development, Aryal B. et al. detected the specific carbonylation of filamin A, heat shock protein 90β, and bifunctional glutamate/proline-tRNA ligase in breast tumor tissues [181]. 

Ros J. et al. have collected information about carbonylated proteins, which are highly relevant in the context of aging-related pathologies, from bacteria and yeast to mammals [182]. As a result, a total of 179 proteins were selected according to their physiological function. Consequently, the authors showed that the proteins involved in cell metabolism and cytoskeleton organization and heat shock proteins were the most significant groups [182]. Next, Dill K.A. et al. have selected 20 human proteins, including transcriptional factor TORs (e.g., Heat shock factor 1 (HSF1)), histones (e.g., H2A Histone Family Member X (H2AFX)), histone-modifying proteins (e.g., Metastasis-associated protein (MTA1)), ribosomal proteins (e.g., Ribosomal protein S6 (RPS6)), and telemetric proteins (e.g., TERF2 Interacting Protein (TERF2IP)), which resulted in higher carbonylation with age and abundancy in older organisms [183]. 

One key feature in the use of protein carbonyls in assessing oxidative damage is the fact that they are chemically stable and can be stored at −80 °C for three months without changes in detectability [184]. The detection and quantification of oxidative protein modifications may be performed using different types of techniques. Global carbonylation can be detected in protein lysates using the derivatization of carbonyl groups with 2,4-dinitrophenylhydrazine (DNPH), which leads to the formation of a 2,4-dinitrophenyl (DNP) hydrazone product. Then, the measurement of protein carbonylation is performed using mass spectrophotometry, immunodetection with DNPH-specific antibodies, an ELISA assay, or immunohistochemistry. The semiquantitative detection of carbonyl content in individual proteins is assessed by one- or two-dimension electrophoresis, followed by western blot [185,186,187]. 

There are many available techniques for the detection of protein carbonyls, but still, it is not clear how protein sequence and structure can affect their susceptibility to oxidative damage.

## 8. Conclusions and Further Perspectives

Here, we discussed recent discoveries on cellular senescence characteristics and their association with age-related disorders. Alterations at the protein expression level impair signaling pathways, which inevitably leads to the dysregulation of organelle communication and disrupts overall cellular homeostasis. However, despite the establishment of a direct correlation between some gene mutations and age-related diseases, causes and effects of aging degeneration remain unclear in many pathological conditions. Uncertainty in the field is also due to the complexity of aging degeneration conditions that occur both at the genomic and at the proteomic level. 

Aberrant proteins can be used as aging biomarkers, and current techniques (such as mass spectrometry and ELISA) can provide precise identifications of protein modifications. However, it is essential to define which specific proteins are the most sensitive to oxidation in different pathologies and why. The identification of oxidized proteins and the definition of their roles in various diseases may promote new avenues of diagnosis and targeted therapeutic approaches. 

For this, the development of new techniques that can identify protein modifications in in vivo models can contribute to the development of therapeutic strategies against various diseases. Furthermore, investigations in this direction can help to detect the degree of oxidative protein damage in an early stage of dysfunction and to address the potential cytotoxic effects of protein aggregate generation in cells.

In particular, protein carbonylation is a well-known marker for oxidative stress and has been detected in various age-related diseases, but its precise contribution to aging degeneration is still unclear, as is the cause and effect of other kinds of degeneration occurring during aging. 

## Figures and Tables

**Table 1:** Signaling pathways and mechanisms involved in aging.

Signaling Pathway	Mechanism	Role in Senescence Context	Ref.
**Genome surveillance signaling**	**DNA repair**	**Normal lifespan control** Mutations in the number of DNA repair genes caused premature aging.	[93]
	**Telomere length-maintaining**	**Replicative lifespan control** Dysfunctions were observed in telomere-mediated syndromes, such as dyskeratosis congenital, idiopathic pulmonary fibrosis, and Alzheimer’s disease.	[31,32,33,94]
	**Tumor-suppressor expressions**	**Promotion of longevity through cancer prevention** Splicing defects of the p53 gene occurred in progeria, vascular aging, and Alzheimer’s disease.	[53]
**Mitochondria and ROS signaling**	**Electron transport**	**Cellular energy control, reactive oxygen species (ROS) production/detoxification, and apoptosis** A reduction of energy and ROS production could extend lifespans while reducing oxidative stress and the formation of carbonylated proteins.	[95]
	**Sirtuin deacetylase activity**	**Regulation of replicative lifespan** In neurodegenerative diseases, sirtuin expression increased, and they acted as neuroprotective molecules in sensing and mitigating ROS. Sirtuin proteins could promote or suppress cancer development.	[78,96,97]
**Hormonal signaling**	**Insulin/IGF-1 activity**	**Growth, remodeling, and aging of tissues** The insulin-like growth factor (IGF) system could play an important role in life processes (cell growth, division, differentiation, apoptosis, aging, and others) by binding with the receptor or activating multiple intracellular signaling cascades. Deregulation of the IGF-1 mechanism was associated with progeria, vascular aging, and Alzheimer’s disease.	[53,98]
	**Transforming growth factor (TGF)-β action**	**Cell growth and proliferation, migration, the regulation of the inflammatory response, wound healing, fibrosis, and cellular apoptosis** Impairment of the TGF-β1 signaling pathway was demonstrated to be specific for brain cells in Alzheimer’s patients, fibrosis, and various types of human cancer, including breast, colon, and renal cancer.	[99,100]
**Metabolic signaling**	**Notch action**	**Embryogenesis, maintenance of tissue specific homeostasis, and stem cell differentiation** The Notch pathway controlled proliferation, migration, the functions of tissue cells, as well as cross-talk between inflammatory cells and the innate immune system. Notch mutations were associated with sporadic Alzheimer’s disease and other neurodegenerative diseases such as Down syndrome, Pick’s and Prion’s disease, and CADASIL (Cerebral Autosomal Dominant Arteriopathy with Subcortical Infarcts and Leucoencephalopathy).	[101,102,103]
	**Wnt action**	**Embryogenesis, cell fate determination, and cell survival** Increased Wnt levels inhibited myogenic differentiation in the elderly. Impaired Notch-TGF-β–Wnt balances in stem cells resulted in the loss of cellular homeostasis.	[104,105]
	**Phosphoinositide 3-kinase (PI3K)/dependent-protein kinase B (AKT)/mTORC2 (PI3K/AKT/mTORC2) cascade**	**Cell proliferation, the regulation of translation, the quality control of proteins, and autophagy** The regulation of pro- and antiaging signaling. The dysregulation of the PI3K–AKT–mTORC2 pathway was strongly associated with tumorigenesis.	[106,107]
	**Serine/threonine-protein kinase (Raf)/ Mitogen-activated protein kinases (MAPK/ERK) cascade**	**Regulation of apoptosis, cell survival, motility, adhesion, proliferation** This pathway has a role in delivering extracellular signals to the nucleus, regulating cellular behavior and longevity.	[108,109,110]
	**Ras/cAMP-dependent protein kinase cascade**	**Regulation of cell survival, replicative senescence, autophagy, and cytoskeleton organization** This cascade regulates caloric restriction and chronological lifespan in yeast. Mutations in Ras resulted in its activation occurring in 1/3 of human tumors (e.g., melanoma, thyroid, colon, and ovarian cancers).	[22,109]

## Abbreviations

ROS	reactive oxygen species
DSB	DNA double-strand break
HR	homologous recombination
NHEJ	nonhomologous end-joining
EDARADD	Edar-associated death domain
TOM1L1	Target of Myb1-like 1 membrane
NPTX2	Neuronal pentraxin II
HAT/KAT	histone/lysine acetyltransferase
HDAC	histone deacetylase
mTOR	target of rapamycin kinase
PI3K	phosphoinositide 3-kinase
AKT	protein kinase B
UPS	ubiquitin–proteasome system
CMA	chaperone-mediated autophagy
DNP	2,4-dinitrophenyl
DNPH	2,4-dinitrophenylhydrazine

## References

[1] Raimundo N., Krisko A. (2018). Cross-organelle communication at the core of longevity. Aging.

[2] Scheibye-Knudsen M., Fang E.F., Croteau D.L., Wilson D.M., Bohr V.A. (2015). Protecting the mitochondrial powerhouse. Trends Cell Biol..

[3] Díaz-Villanueva J.F., Díaz-Molina R., García-González V. (2015). Protein Folding and Mechanisms of Proteostasis. Int. J. Mol. Sci..

[4] Fedarko N.S. (2011). The Biology of Aging and Frailty. Clin. Geriatr. Med..

[5] Jung Y., Brack A.S. (2014). Cellular Mechanisms of Somatic Stem Cell Aging. Curr. Top. Dev. Biol..

[6] Belikov A.V. (2019). Age-related diseases as vicious cycles. Ageing Res. Rev..

[7] Lopez-Otin C., Blasco M.A., Partridge L., Serrano M., Kroemer G. (2013). The hallmarks of aging. Cell.

[8] Moeller M., Hirose M., Mueller S., Roolf C., Baltrusch S., Ibrahim S., Junghanss C., Wolkenhauer O., Jaster R., Köhling R. (2014). Inbred mouse strains reveal biomarkers that are pro-longevity, antilongevity or role switching. Aging Cell.

[9] Pincus Z., Slack F.J. (2010). Developmental biomarkers of aging in C. elegans. Dev. Dyn..

[10] Gire V., Dulic V. (2015). Senescence from G2 arrest, revisited. Cell Cycle.

[11] Kaeberlein M., Powers R.W., Steffen K.K., Westman E.A., Hu D., Dang N., Kerr E.O., Kirkland K.T., Fields S., Kennedy B.K. (2005). Regulation of Yeast Replicative Life Span by TOR and Sch9 in Response to Nutrients. Science.

[12] He C., Zhou C., Kennedy B.K. (2018). The yeast replicative aging model. Biochim. Biophys. Acta.

[13] Kaeberlein M. (2010). Lessons on longevity from budding yeast. Nature.

[14] Katajisto P., Dohla J., Chaffer C.L., Pentinmikko N., Marjanovic N., Iqbal S., Zoncu R., Chen W., Weinberg R.A., Sabatini D.M. (2015). Stem cells. Asymmetric apportioning of aged mitochondria between daughter cells is required for stemness. Science.

[15] Van Deursen J.M. (2014). The role of senescent cells in ageing. Nature.

[16] Kato D., Miyazawa K., Ruas M., Starborg M., Wada I., Oka T., Sakai T., Peters G., Hara E. (1998). Features of replicative senescence induced by direct addition of antennapedia-p16INK4A fusion protein to human diploid fibroblasts. FEBS Lett..

[17] Lee H., Adams W.J., Alford P.W., McCain M.L., Feinberg A.W., Sheehy S.P., Goss J.A., Parker K.K. (2015). Cytoskeletal prestress regulates nuclear shape and stiffness in cardiac myocytes. Exp. Biol. Med..

[18] Baird N.A., Douglas P.M., Simic M.S., Grant A.R., Moresco J.J., Wolff S.C., Yates J.R., Manning G., Dillin A. (2014). HSF-1-mediated cytoskeletal integrity determines thermotolerance and life span. Science.

[19] Perkins A.D., Lee M.J.J., Tanentzapf G. (2014). The systematic identification of cytoskeletal genes required for. Sci. Data.

[20] Rodrigues A.J., do Carmo Costa M., Silva T.L., Ferreira D., Bajanca F., Logarinho E., Maciel P. (2010). Absence of ataxin-3 leads to cytoskeletal disorganization and increased cell death. Biochim. Biophys. Acta.

[21] Gourlay C.W., Ayscough K.R. (2018). The actin cytoskeleton in ageing and apoptosis. FEMS Yeast Res..

[22] Gong C.X., Iqbal K. (2008). Hyperphosphorylation of microtubule-associated protein tau: A promising therapeutic target for Alzheimer disease. Curr. Med. Chem..

[23] Prachar J. (2003). Intimate contacts of mitochondria with nuclear envelope as a potential energy gateway for nucleo-cytoplasmic mRNA transport. Gen. Physiol. Biophys..

[24] Rowe L.A., Degtyareva N., Doetsch P.W. (2008). DNA Damage-induced Reactive Oxygen Species (ROS) Stress Response in Saccharomyces cerevisiae. Free Radic. Biol. Med..

[25] Langie S.A., Koppen G., Desaulniers D., Al-Mulla F., Al-Temaimi R., Amedei A., Azqueta A., Bisson W.H., Brown D.G., Brunborg G. (2015). Causes of genome instability: The effect of low dose chemical exposures in modern society. Carcinogenesis.

[26] Lieber M.R. (2010). The mechanism of double-strand DNA break repair by the nonhomologous DNA end-joining pathway. Annu. Rev. Biochem..

[27] David S.S., O’Shea V.L., Kundu S. (2007). Base Excision Repair of Oxidative DNA Damage. Nature.

[28] Schärer O.D. (2013). Nucleotide Excision Repair in Eukaryotes. Cold Spring Harb. Perspect. Biol..

[29] Jiricny J. (2013). Postreplicative Mismatch Repair. Cold Spring Harb. Perspect. Biol..

[30] O’Sullivan R.J., Karlseder J. (2010). Telomeres: Protecting chromosomes against genome instability. Nat. Rev. Mol. Cell Biol..

[31] Gramatges M.M., Bertuch A.A. (2013). Short telomeres: From dyskeratosis congenita to sporadic aplastic anemia and malignancy. Transl. Res. J. Lab. Clin. Med..

[32] Alder J.K., Chen J.J., Lancaster L., Danoff S., Su S.C., Cogan J.D., Vulto I., Xie M., Qi X., Tuder R.M. (2008). Short telomeres are a risk factor for idiopathic pulmonary fibrosis. Proc. Natl. Acad. Sci. USA.

[33] Cai Z., Yan L.J., Ratka A. (2013). Telomere shortening and Alzheimer’s disease. Neuromol. Med..

[34] Bannister A.J., Kouzarides T. (2011). Regulation of chromatin by histone modifications. Cell Res..

[35] Liyanage V.R.B., Jarmasz J.S., Murugeshan N., Del Bigio M.R., Rastegar M., Davie J.R. (2014). DNA Modifications: Function and Applications in Normal and Disease States. Biology.

[36] Jin B., Li Y., Robertson K.D. (2011). DNA Methylation: Superior or Subordinate in the Epigenetic Hierarchy?. Genes Cancer.

[37] Horvath S. (2013). DNA methylation age of human tissues and cell types. Genome Biol..

[38] Johnson A.A., Akman K., Calimport S.R., Wuttke D., Stolzing A., de Magalhães J.P. (2012). The Role of DNA Methylation in Aging, Rejuvenation, and Age-Related Disease. Rejuvenation Res..

[39] Headon D.J., Emmal S.A., Ferguson B.M., Tucker A.S., Justice M.J., Sharpe P.T., Zonana J., Overbeek P.A. (2001). Gene defect in ectodermal dysplasia implicates a death domain adapter in development. Nature.

[40] Franco M., Furstoss O., Simon V., Benistant C., Hong W.J., Roche S. (2006). The Adaptor Protein Tom1L1 Is a Negative Regulator of Src Mitogenic Signaling Induced by Growth Factors. Mol. Cell. Biol..

[41] Schaukowitch K., Reese A.L., Kim S.K., Kilaru G., Joo J.Y., Kavalali E.T., Kim T.K. (2017). An intrinsic transcriptional program underlying synaptic scaling during activity suppression. Cell Rep..

[42] Seto E., Yoshida M. (2014). Erasers of Histone Acetylation: The Histone Deacetylase Enzymes. Cold Spring Harb. Perspect. Biol..

[43] Madeo F., Zimmermann A., Maiuri M.C., Kroemer G. (2015). Essential role for autophagy in life span extension. J. Clin. Investig..

[44] Chang K.T., Min K.T. (2002). Regulation of lifespan by histone deacetylase. Ageing Res. Rev..

[45] Bhullar K.S., Hubbard B.P. (2015). Lifespan and healthspan extension by resveratrol. Biochim. Biophys. Acta (BBA) Mol. Basis Dis..

[46] Ryan J.M., Cristofalo V.J. (1972). Histone acetylation during aging of human cells in culture. Biochem. Biophys. Res. Commun..

[47] De Magalhães J.P., Curado J., Church G.M. (2009). Meta-analysis of age-related gene expression profiles identifies common signatures of aging. Bioinformatics.

[48] Frenk S., Houseley J. (2018). Gene expression hallmarks of cellular ageing. Biogerontology.

[49] Belfort M. (2017). Mobile self-splicing introns and inteins as environmental sensors. Curr. Opin. Microbiol..

[50] Rudan M., Bou Dib P., Musa M., Kanunnikau M., Sobocanec S., Rueda D., Warnecke T., Krisko A. (2018). Normal mitochondrial function in Saccharomyces cerevisiae has become dependent on inefficient splicing. eLife.

[51] Li H., Wang Z., Ma T., Wei G., Ni T. (2013). Alternative splicing in aging and age-related diseases. Cold Spring Harb Perspect Biol..

[52] Lu T., Pan Y., Kao S.-Y., Li C., Kohane I., Chan J., Yankner B.A. (2004). Gene regulation and DNA damage in the ageing human brain. Nature.

[53] Deschênes M., Chabot B. (2017). The emerging role of alternative splicing in senescence and aging. Aging Cell.

[54] Anisimova A.S., Alexandrov A.I., Makarova N.E., Gladyshev V.N., Dmitriev S.E. (2018). Protein synthesis and quality control in aging. Aging.

[55] Rogers A.N., Chen D., McColl G., Czerwieniec G., Felkey K., Gibson B.W., Hubbard A., Melov S., Lithgow G.J., Kapahi P. (2011). Life span extension via eIF4G inhibition is mediated by posttranscriptional remodeling of stress response gene expression in C. elegans. Cell Metab..

[56] Luce M.C., Bunn C.L. (1989). Decreased accuracy of protein synthesis in extracts from aging human diploid fibroblasts. Exp. Gerontol..

[57] Li M., Xiao Z.Q., Chen Z.C., Li J.L., Li C., Zhang P.F., Li M.Y. (2007). Proteomic analysis of the aging-related proteins in human normal colon epithelial tissue. J. Biochem. Mol. Biol..

[58] Yang S., Liu T., Li S., Zhang X., Ding Q., Que H., Yan X., Wei K., Liu S. (2008). Comparative proteomic analysis of brains of naturally aging mice. Neuroscience.

[59] Waldera-Lupa D.M., Kalfalah F., Florea A.M., Sass S., Kruse F., Rieder V., Tigges J., Fritsche E., Krutmann J., Busch H. (2014). Proteome-wide analysis reveals an age-associated cellular phenotype of in situ aged human fibroblasts. Aging.

[60] Wang K.C., Chang H.Y. (2011). Molecular mechanisms of long noncoding RNAs. Mol. Cell.

[61] Gomes A.Q., Nolasco S., Soares H. (2013). Non-Coding RNAs: Multi-Tasking Molecules in the Cell. Int. J. Mol. Sci..

[62] Fernandes J.C., Acuña S.M., Aoki J.I., Floeter-Winter L.M., Muxel S.M. (2019). Long non-coding RNAs in the regulation of gene expression: Physiology and disease. Non-Coding RNA.

[63] Bartel D.P. (2004). MicroRNAs: Genomics, biogenesis, mechanism, and function. Cell.

[64] Garg D., Cohen S.M. (2014). miRNAs and aging: A genetic perspective. Ageing Res. Rev..

[65] Sen C.K., Roy S. (2012). MicroRNA 21 in tissue injury and inflammation. Cardiovasc. Res..

[66] Hooten N.N., Fitzpatrick M., Wood W.H., De S., Ejiogu N., Zhang Y., Mattison J.A., Becker K.G., Zonderman A.B., Evans M.K. (2013). Age-related changes in microRNA levels in serum. Aging.

[67] Fernandes D.P., Bitar M., Jacobs F., Barry G. (2018). Long Non-Coding RNAs in Neuronal Aging. Non-Coding RNA.

[68] Riva P., Ratti A., Venturin M. (2016). The Long Non-Coding RNAs in Neurodegenerative Diseases: Novel Mechanisms of Pathogenesis. Curr. Alzheimer Res..

[69] Sahin E., DePinho R.A. (2012). Axis of ageing: Telomeres, p53 and mitochondria. Nat. Rev. Mol. Cell Biol..

[70] Samuels D.C., Li C., Li B., Song Z., Torstenson E., Boyd Clay H., Rokas A., Thornton-Wells T.A., Moore J.H., Hughes T.M. (2013). Recurrent tissue-specific mtDNA mutations are common in humans. PLoS Genet..

[71] Sun N., Youle R.J., Finkel T. (2016). The Mitochondrial Basis of Aging. Mol. Cell.

[72] Harman D. (1972). The biologic clock: The mitochondria?. J. Am. Geriatr. Soc..

[73] Khrapko K., Vijg J. (2009). Mitochondrial DNA mutations and aging: Devils in the details?. Trends Genet. TIG.

[74] Park C.B., Larsson N.-G. (2011). Mitochondrial DNA mutations in disease and aging. J. Cell Biol..

[75] Pinto M., Moraes C.T. (2015). Mechanisms linking mtDNA damage and aging. Free Radic. Biol. Med..

[76] Fox R., Kim H.-S., Reddick R.L., Kujoth G.C., Prolla T.A., Tsutsumi S., Wada Y., Smithies O., Maeda N. (2011). Mitochondrial DNA polymerase editing mutation, PolgD257A, reduces the diabetic phenotype of Akita male mice by suppressing appetite. Proc. Natl. Acad. Sci. USA.

[77] Wallace D.C. (1999). Mitochondrial diseases in man and mouse. Science.

[78] Weir H.J., Murray T.K., Kehoe P.G., Love S., Verdin E.M., O’Neill M.J., Lane J.D., Balthasar N. (2012). CNS SIRT3 expression is altered by reactive oxygen species and in Alzheimer’s disease. PLoS ONE.

[79] Santos J., Leitao-Correia F., Sousa M.J., Leao C. (2016). Dietary Restriction and Nutrient Balance in Aging. Oxidative Med. Cell. Longev..

[80] Renaville R., Hammadi M., Portetelle D. (2002). Role of the somatotropic axis in the mammalian metabolism. Domest. Anim. Endocrinol..

[81] Milman S., Huffman D.M., Barzilai N. (2016). The Somatotropic Axis in Human Aging: Framework for the Current State of Knowledge and Future Research. Cell Metab..

[82] Van Heemst D. (2010). Insulin, IGF-1 and longevity. Aging Dis..

[83] Kenyon C. (2011). The first long-lived mutants: Discovery of the insulin/IGF-1 pathway for ageing. Philos. Trans. R. Soc. B Biol. Sci..

[84] Laplante M., Sabatini D.M. (2012). mTOR signaling in growth control and disease. Cell.

[85] Ruetenik A., Barrientos A. (2015). Dietary restriction, mitochondrial function and aging: From yeast to humans. Biochim. Biophys. Acta.

[86] Katewa S.D., Kapahi P. (2010). Dietary restriction and aging, 2009. Aging Cell.

[87] Zoncu R., Efeyan A., Sabatini D.M. (2011). mTOR: From growth signal integration to cancer, diabetes and ageing. Nat. Rev. Mol. cell Biol..

[88] Mihaylova M.M., Shaw R.J. (2011). The AMPK signaling pathway coordinates cell growth, autophagy and metabolism. Nat. Cell Biol..

[89] Salminen A., Kaarniranta K. (2012). AMP-activated protein kinase (AMPK) controls the aging process via an integrated signaling network. Ageing Res. Rev..

[90] Kaeberlein M., McVey M., Guarente L. (1999). The SIR2/3/4 complex and SIR2 alone promote longevity in Saccharomyces cerevisiae by two different mechanisms. Genes Dev..

[91] Jing H., Lin H. (2015). Sirtuins in epigenetic regulation. Chem. Rev..

[92] North B.J., Verdin E. (2004). Sirtuins: Sir2-related NAD-dependent protein deacetylases. Genome Biol..

[93] Maynard S., Fang E.F., Scheibye-Knudsen M., Croteau D.L., Bohr V.A. (2015). DNA damage, DNA repair, aging, and neurodegeneration. Cold Spring Harb. Perspect. Med..

[94] Rudolph K.L., Chang S., Lee H.-W., Blasco M., Gottlieb G.J., Greider C., DePinho R.A. (1999). Longevity, stress response, and cancer in aging telomerase-deficient mice. Cell.

[95] Monaghan R.M., Barnes R.G., Fisher K., Andreou T., Rooney N., Poulin G.B., Whitmarsh A.J. (2015). A nuclear role for the respiratory enzyme CLK-1 in regulating mitochondrial stress responses and longevity. Nat. Cell Biol..

[96] Longo V.D., Kennedy B.K. (2006). Sirtuins in aging and age-related disease. Cell.

[97] Carafa V., Altucci L., Nebbioso A. (2019). Dual Tumor Suppressor and Tumor Promoter Action of Sirtuins in Determining Malignant Phenotype. Front. Pharmacol..

[98] Holzenberger M., Dupont J., Ducos B., Leneuve P., Géloën A., Even P.C., Cervera P., Le Bouc Y. (2003). IGF-1 receptor regulates lifespan and resistance to oxidative stress in mice. Nature.

[99] Massagué J., Chen Y.-G. (2000). Controlling TGF-β signaling. Genes Dev..

[100] Colak S., Ten Dijke P. (2017). Targeting TGF-beta Signaling in Cancer. Trends Cancer.

[101] Carlson M.E., Conboy I.M. (2007). Regulating the Notch pathway in embryonic, adult and old stem cells. Curr. Opin. Pharmacol..

[102] Weinmaster G. (1997). The ins and outs of notch signaling. Mol. Cell. Neurosci..

[103] Polychronidou E., Vlachakis D., Vlamos P., Baumann M., Kossida S. (2015). Notch signaling and ageing. Adv. Exp. Med. Biol..

[104] Carlson M.E., Silva H.S., Conboy I.M. (2008). Aging of signal transduction pathways, and pathology. Exp. Cell Res..

[105] Moon R. (2004). Wnt and β-catenin signaling: Diseases and therapies. Nat. Rev. Genet..

[106] Tan P., Wang Y.J., Li S., Wang Y., He J.Y., Chen Y.Y., Deng H.Q., Huang W., Zhan J.K., Liu Y.S. (2016). The PI3K/Akt/mTOR pathway regulates the replicative senescence of human VSMCs. Mol. Cell. Biochem..

[107] Johnson S.C., Rabinovitch P.S., Kaeberlein M. (2013). mTOR is a key modulator of ageing and age-related disease. Nature.

[108] Kolch W. (2005). Coordinating ERK/MAPK signaling through scaffolds and inhibitors. Nat. Rev. Mol. Cell Biol..

[109] Slack C. (2017). Ras signaling in aging and metabolic regulation. Nutr. Healthy Aging.

[110] Zou J., Lei T., Guo P., Yu J., Xu Q., Luo Y., Ke R., Huang D. (2019). Mechanisms shaping the role of ERK1/2 in cellular senescence. Mol. Med. Rep..

[111] Jazwinski S.M. (2014). The retrograde response: A conserved compensatory reaction to damage from within and from without. Prog. Mol. Biol. Transl. Sci..

[112] Heintz C., Doktor T.K., Lanjuin A., Escoubas C., Zhang Y., Weir H.J., Dutta S., Silva-Garcia C.G., Bruun G.H., Morantte I. (2017). Splicing factor 1 modulates dietary restriction and TORC1 pathway longevity in C. elegans. Nature.

[113] Baker M.J., Palmer C.S., Stojanovski D. (2014). Mitochondrial protein quality control in health and disease. Br. J. Pharmacol..

[114] Pellegrino M.W., Nargund A.M., Haynes C.M. (2013). Signaling the mitochondrial unfolded protein response. Biochim. Biophys. Acta.

[115] Perić M., Lovrić A., Šarić A., Musa M., Bou Dib P., Rudan M., Nikolić A., Sobočanec S., Mikecin A., Dennerlein S. (2017). TORC1-mediated sensing of chaperone activity alters glucose metabolism and extends lifespan. Aging Cell.

[116] Murley A., Nunnari J. (2016). The emerging network of mitochondria-organelle contacts. Mol. Cell.

[117] Rusinol A.E., Cui Z., Chen M.H., Vance J.E. (1994). A unique mitochondria-associated membrane fraction from rat liver has a high capacity for lipid synthesis and contains pre-Golgi secretory proteins including nascent lipoproteins. J. Biol. Chem..

[118] Leite M.F., Thrower E.C., Echevarria W., Koulen P., Hirata K., Bennett A.M., Ehrlich B.E., Nathanson M.H. (2003). Nuclear and cytosolic calcium are regulated independently. Proc. Natl. Acad. Sci. USA.

[119] Lasorsa F.M., Pinton P., Palmieri L., Scarcia P., Rottensteiner H., Rizzuto R., Palmieri F. (2008). Peroxisomes as novel players in cell calcium homeostasis. J. Biol. Chem..

[120] Wong A.K., Capitanio P., Lissandron V., Bortolozzi M., Pozzan T., Pizzo P. (2013). Heterogeneity of Ca2+ handling among and within Golgi compartments. J. Mol. Cell Biol..

[121] Behringer E.J., Segal S.S. (2017). Impact of aging on calcium signaling and membrane potential in endothelium of resistance arteries: A role for mitochondria. J. Gerontol. Ser. A Biomed. Sci. Med Sci..

[122] Puzianowska-Kuznicka M., Kuznicki J. (2009). The ER and ageing II: Calcium homeostasis. Ageing Res. Rev..

[123] Chandhok G., Lazarou M., Neumann B. (2018). Structure, function, and regulation of mitofusin-2 in health and disease. Biol. Rev. Camb. Philos. Soc..

[124] Janikiewicz J., Szymański J., Malinska D., Patalas-Krawczyk P., Michalska B., Duszyński J., Giorgi C., Bonora M., Dobrzyn A., Wieckowski M.R. (2018). Mitochondria-associated membranes in aging and senescence: Structure, function, and dynamics. Cell Death Dis..

[125] Sebastian D., Hernandez-Alvarez M.I., Segales J., Sorianello E., Munoz J.P., Sala D., Waget A., Liesa M., Paz J.C., Gopalacharyulu P. (2012). Mitofusin 2 (Mfn2) links mitochondrial and endoplasmic reticulum function with insulin signaling and is essential for normal glucose homeostasis. Proc. Natl. Acad. Sci. USA.

[126] Shankar J., Kojic L.D., St-Pierre P., Wang P.T., Fu M., Joshi B., Nabi I.R. (2013). Raft endocytosis of AMF regulates mitochondrial dynamics through Rac1 signaling and the Gp78 ubiquitin ligase. J. Cell Sci..

[127] Fang L., Moore X.L., Gao X.M., Dart A.M., Lim Y.L., Du X.J. (2007). Down-regulation of mitofusin-2 expression in cardiac hypertrophy in vitro and in vivo. Life Sci..

[128] Bach D., Naon D., Pich S., Soriano F.X., Vega N., Rieusset J., Laville M., Guillet C., Boirie Y., Wallberg-Henriksson H. (2005). Expression of Mfn2, the Charcot-Marie-Tooth neuropathy type 2A gene, in human skeletal muscle: Effects of type 2 diabetes, obesity, weight loss, and the regulatory role of tumor necrosis factor alpha and interleukin-6. Diabetes.

[129] Area-Gomez E., del Carmen Lara Castillo M., Tambini M.D., Guardia-Laguarta C., de Groof A.J.C., Madra M., Ikenouchi J., Umeda M., Bird T.D., Sturley S.L. (2012). Upregulated function of mitochondria-associated ER membranes in Alzheimer disease. EMBO J..

[130] Sala A.J., Bott L.C., Morimoto R.I. (2017). Shaping proteostasis at the cellular, tissue, and organismal level. J. Cell Biol..

[131] Koga H., Kaushik S., Cuervo A.M. (2011). Protein Homeostasis and Aging: The importance of exquisite quality control. Ageing Res. Rev..

[132] Sampaio-Marques B., Ludovico P. (2018). Linking cellular proteostasis to yeast longevity. FEMS Yeast Res..

[133] Andréasson C., Ott M., Büttner S. (2019). Mitochondria orchestrate proteostatic and metabolic stress responses. EMBO Rep..

[134] Kaushik S., Cuervo A.M. (2015). Proteostasis and aging. Nat. Med..

[135] Nedelsky N., Todd P.K., Taylor J.P. (2008). Autophagy and the ubiquitin-proteasome system: Collaborators in neuroprotection. Biochim. Biophys. Acta.

[136] Klaips C.L., Jayaraj G.G., Hartl F.U. (2018). Pathways of cellular proteostasis in aging and disease. J. Cell Biol..

[137] Taylor J.P., Hardy J., Fischbeck K.H. (2002). Toxic proteins in neurodegenerative disease. Science.

[138] Eisele Y.S., Monteiro C., Fearns C., Encalada S.E., Wiseman R.L., Powers E.T., Kelly J.W. (2015). Targeting Protein Aggregation for the Treatment of Degenerative Diseases. Nat. Rev. Drug Discov..

[139] von Mikecz A., Chen M., Rockel T., Scharf A. (2008). The nuclear ubiquitin-proteasome system: Visualization of proteasomes, protein aggregates, and proteolysis in the cell nucleus. Methods Mol. Biol..

[140] Rao R.V., Bredesen D.E. (2004). Misfolded proteins, endoplasmic reticulum stress and neurodegeneration. Curr. Opin. Cell Biol..

[141] Oslowski C.M., Urano F. (2011). Measuring ER stress and the unfolded protein response using mammalian tissue culture system. Methods Enzymol..

[142] Zhong J., Alan P.E., Alice L. (2013). Endoplasmic Reticulum (ER) Stress in the Pathogenesis of Type 1 Diabetes. Type 1 Diabetes.

[143] Lindholm D., Wootz H., Korhonen L. (2006). ER stress and neurodegenerative diseases. Cell Death Differ..

[144] Deretic V. (2010). A Master Conductor for Aggregate Clearance by Autophagy. Dev. Cell.

[145] Eskelinen E.-L. (2013). Macroautophagy in Mammalian Cells. https://www.ncbi.nlm.nih.gov/books/NBK6211/.

[146] Bandyopadhyay U., Kaushik S., Varticovski L., Cuervo A.M. (2008). The chaperone-mediated autophagy receptor organizes in dynamic protein complexes at the lysosomal membrane. Mol. Cell. Biol..

[147] Mizushima N., Levine B. (2010). Autophagy in mammalian development and differentiation. Nat. Cell Biol..

[148] Wang C.W., Klionsky D.J. (2003). The Molecular Mechanism of Autophagy. Mol. Med..

[149] Bejarano E., Cuervo A.M. (2010). Chaperone-Mediated Autophagy. Proc. Am. Thorac. Soc..

[150] Hipp M.S., Kasturi P., Hartl F.U. (2019). The proteostasis network and its decline in ageing. Nat. Rev. Mol. Cell Biol..

[151] Moreau K.L., King J.A. (2012). Protein misfolding and aggregation in cataract disease and prospects for prevention. Trends Mol. Med..

[152] Lashuel H.A., Hartley D., Petre B.M., Walz T., Lansbury Jr P.T. (2002). Neurodegenerative disease: Amyloid pores from pathogenic mutations. Nature.

[153] Milanesi L., Sheynis T., Xue W.-F., Orlova E.V., Hellewell A.L., Jelinek R., Hewitt E.W., Radford S.E., Saibil H.R. (2012). Direct three-dimensional visualization of membrane disruption by amyloid fibrils. Proc. Natl. Acad. Sci. USA.

[154] Cai Z., Yan L.J. (2013). Protein Oxidative Modifications: Beneficial Roles in Disease and Health. J. Biochem. Pharmacol. Res..

[155] Fedorova M., Bollineni R.C., Hoffmann R. (2014). Protein carbonylation as a major hallmark of oxidative damage: Update of analytical strategies. Mass Spectrom. Rev..

[156] Semchyshyn H.M. (2014). Reactive carbonyl species in vivo: Generation and dual biological effects. Sci. World J..

[157] Riley P.A. (1994). Free radicals in biology: Oxidative stress and the effects of ionizing radiation. Int. J. Radiat. Biol..

[158] Negre-Salvayre A., Coatrieux C., Ingueneau C., Salvayre R. (2008). Advanced lipid peroxidation end products in oxidative damage to proteins. Potential role in diseases and therapeutic prospects for the inhibitors. Br. J. Pharmacol..

[159] Adams S., Green P., Claxton R., Simcox S., Williams M.V., Walsh K., Leeuwenburgh C. (2001). Reactive carbonyl formation by oxidative and non-oxidative pathways. Front. Biosci..

[160] Reznick A.Z., Packer L. (1994). [38] Oxidative damage to proteins: Spectrophotometric method for carbonyl assay. Methods in Enzymology.

[161] Weber D., Davies M.J., Grune T. (2015). Determination of protein carbonyls in plasma, cell extracts, tissue homogenates, isolated proteins: Focus on sample preparation and derivatization conditions. Redox Biol..

[162] Levine R.L., Stadtman E.R. (2001). Oxidative modification of proteins during aging. Exp. Gerontol..

[163] Gianni P., Jan K.J., Douglas M.J., Stuart P.M., Tarnopolsky M.A. (2004). Oxidative stress and the mitochondrial theory of aging in human skeletal muscle. Exp. Gerontol..

[164] Floyd R.A., Hensley K. (2002). Oxidative stress in brain aging: Implications for therapeutics of neurodegenerative diseases. Neurobiol. Aging.

[165] Mutlu-Türkoğlu Ü., İlhan E., Öztezcan S., Kuru A., Aykaç-Toker G., Uysal M. (2003). Age-related increases in plasma malondialdehyde and protein carbonyl levels and lymphocyte DNA damage in elderly subjects. Clin. Biochem..

[166] Forster M.J., Sohal B.H., Sohal R.S. (2000). Reversible effects of long-term caloric restriction on protein oxidative damage. J. Gerontol. Ser. A Biol. Sci. Med Sci..

[167] Pickering A.M., Davies K.J. (2012). Degradation of damaged proteins: The main function of the 20S proteasome. Prog. Mol. Biol. Transl. Sci..

[168] Tanase M., Urbanska A.M., Zolla V., Clement C.C., Huang L., Morozova K., Follo C., Goldberg M., Roda B., Reschiglian P. (2016). Role of carbonyl modifications on aging-associated protein aggregation. Sci. Rep..

[169] Telci A., Çakatay U., Salman S., Satman I.l., Sivas A. (2000). Oxidative protein damage in early stage Type 1 diabetic patients. Diabetes Res. Clin. Pract..

[170] Butterfield D.A., Lauderback C.M. (2002). Lipid peroxidation and protein oxidation in Alzheimer’s disease brain: Potential causes and consequences involving amyloid β-peptide-associated free radical oxidative stress. Free Radic. Biol. Med..

[171] Túnez I., Sánchez-López F., Agüera E., Fernández-Bolaños R., Sánchez F.M., Tasset-Cuevas I. (2011). Important role of oxidative stress biomarkers in Huntington’s disease. J. Med. Chem..

[172] Shaw P.J., Ince P.G., Falkous G., Mantle D. (1995). Oxidative damage to protein in sporadic motor neuron disease spinal cord. Ann. Neurol. Off. J. Am. Neurol. Assoc. Child Neurol. Soc..

[173] Katerji M., Filippova M., Duerksen-Hughes P. (2019). Approaches and Methods to Measure Oxidative Stress in Clinical Samples: Research Applications in the Cancer Field. Oxidative Med. Cell. Longev..

[174] Aydin B., Yagci R., Yılmaz F.M., Erdurmus M., Karadağ R., Keskin U., Durmus M., Yigitoglu R. (2009). Prevention of selenite-induced cataractogenesis by N-acetylcysteine in rats. Curr. Eye Res..

[175] Czerwińska J., Poznański J., Dębski J., Bukowy Z., Bohr V.A., Tudek B., Speina E. (2014). Catalytic activities of Werner protein are affected by adduction with 4-hydroxy-2-nonenal. Nucleic Acids Res..

[176] Range S., Dunster C., Knox A., Kelly F. (1999). Treatment of pulmonary exacerbations of cystic fibrosis leads to improved antioxidant status. Eur. Respir. J..

[177] Rodrigo R., Libuy M., Feliú F., Hasson D. (2013). Oxidative stress-related biomarkers in essential hypertension and ischemia-reperfusion myocardial damage. Dis. Markers.

[178] Alam Z.I., Daniel S.E., Lees A.J., Marsden D.C., Jenner P., Halliwell B. (1997). A generalised increase in protein carbonyls in the brain in Parkinson’s but not incidental Lewy body disease. J. Neurochem..

[179] Aksenov M., Aksenova M., Butterfield D., Geddes J., Markesbery W. (2001). Protein oxidation in the brain in Alzheimer’s disease. Neuroscience.

[180] Ferrante R.J., Browne S.E., Shinobu L.A., Bowling A.C., Baik M.J., MacGarvey U., Kowall N.W., Brown R.H., Beal M.F. (1997). Evidence of increased oxidative damage in both sporadic and familial amyotrophic lateral sclerosis. J. Neurochem..

[181] Aryal B., Rao V.A. (2018). Specific protein carbonylation in human breast cancer tissue compared to adjacent healthy epithelial tissue. PLoS ONE.

[182] Cabiscol E., Tamarit J., Ros J. (2014). Protein carbonylation: Proteomics, specificity and relevance to aging. Mass Spectrom. Rev..

[183] De Graff A.M., Hazoglou M.J., Dill K.A. (2016). Highly Charged Proteins: The Achilles’ Heel of Aging Proteomes. Structure.

[184] Griffiths H.R. (2000). Antioxidants and protein oxidation. Free Radic. Res..

[185] Wehr N.B., Levine R.L. (2013). Quantification of protein carbonylation. Methods Mol. Biol..

[186] Augustyniak E., Adam A., Wojdyla K., Rogowska-Wrzesinska A., Willetts R., Korkmaz A., Atalay M., Weber D., Grune T., Borsa C. (2015). Validation of protein carbonyl measurement: A multi-centre study. Redox Biol..

[187] Rodríguez-García A., Morales M.L., Garrido-García V., García-Baquero I., Leivas A., Carreño-Tarragona G., Sánchez R., Arenas A., Cedena T., Ayala R.M. (2019). Protein Carbonylation in Patients with Myelodysplastic Syndrome: An Opportunity for Deferasirox Therapy. Antioxidants.

